# CCL2 Is Associated with a Faster Rate of Cognitive Decline during Early Stages of Alzheimer's Disease

**DOI:** 10.1371/journal.pone.0030525

**Published:** 2012-01-30

**Authors:** Karin Westin, Peder Buchhave, Henrietta Nielsen, Lennart Minthon, Sabina Janciauskiene, Oskar Hansson

**Affiliations:** 1 Clinical Memory Research Unit, Department of Clinical Sciences Malmö, Lund University, Malmö, Sweden; 2 Neuropsychiatric Clinic, Skåne University Hospital, Malmö, Sweden; 3 Molecular Memory Research Unit, Department of Clinical Sciences Malmö, Lund University, Skåne University Hospital, Malmö, Sweden; 4 Hannover Medical School, Hannover, Germany; Nathan Kline Institute and New York University School of Medicine, United States of America

## Abstract

Chemokine (C-C motif) receptor 2 (CCR2)-signaling can mediate accumulation of microglia at sites affected by neuroinflammation. CCR2 and its main ligand CCL2 (MCP-1) might also be involved in the altered metabolism of beta-amyloid (Aβ) underlying Alzheimer's disease (AD). We therefore measured the levels of CCL2 and three other CCR2 ligands, i.e. CCL11 (eotaxin), CCL13 (MCP-4) and CCL26 (eotaxin-3), in the cerebrospinal fluid (CSF) and plasma of 30 controls and 119 patients with mild cognitive impairment (MCI) at baseline. During clinical follow-up 52 MCI patients were clinically stable for five years, 47 developed AD (i.e. cases with prodromal AD at baseline) and 20 developed other dementias. Only CSF CCL26 was statistically significantly elevated in patients with prodromal AD when compared to controls (p = 0.002). However, in patients with prodromal AD, the CCL2 levels in CSF at baseline correlated with a faster cognitive decline during follow-up (*r*_s_ = 0.42, p = 0.004). Furthermore, prodromal AD patients in the highest tertile of CSF CCL2 exhibited a significantly faster cognitive decline (p<0.001) and developed AD dementia within a shorter time period (p<0.003) compared to those in the lowest tertile. Finally, in the entire MCI cohort, CSF CCL2 could be combined with CSF Tau, P-tau and Aβ42 to predict both future conversion to AD and the rate of cognitive decline. If these results are corroborated in future studies, CCL2 in CSF could be a candidate biomarker for prediction of future disease progression rate in prodromal AD. Moreover, CCR2-related signaling pathways might be new therapeutic targets for therapies aiming at slowing down the disease progression rate of AD.

## Introduction

Alzheimer's disease (AD) is the most common cause of dementia in the elderly population. Preceding onset of dementia, patients typically exhibit milder symptoms of cognitive decline, so called mild cognitive impairment (MCI). However, MCI is a heterogeneous syndrome and does not exclusively comprise cases with prodromal AD (i.e. individuals with AD who have not yet developed dementia), but also patients with stable MCI or patients affected by early stages of other types of dementia disorders than AD [1].

AD is characterized by the accumulation of β-amyloid (Aβ) and tau into senile plaques and neurofibrillary tangles, respectively [2], [3]. There is evidence suggesting that intracerebral inflammation, including microgliosis, can play a significant role in the development of AD [4]. Senile plaques are often surrounded by clusters of reactive astrocytes and microglia [4], [5]. Aggregated Aβ can activate glial cells, which then may clear Aβ. However, activated glia cells release inflammatory mediators in this process, including chemokines, that might contribute to neuronal dysfunction and cell death [4], [5], [6]. The chemokine C-C motif ligand 2 (CCL2; also called monocyte chemotactic protein-1 (MCP-1)), which is produced by neurons and glial cells, activates the CCR2-receptor and can induce chemotaxis of monocytes and microglia, contributing to pathological microgliosis [7], [8]. Interestingly, several studies have shown that CCL2-signalling can exacerbate Aβ pathology in animal models of AD [9], [10]. For example, Yamamoto and colleagues have demonstrated that transgenic AD mice expressing mutant APP exhibit increased Aβ pathology when also overexpressing CCL2 [9].

Biochemical changes in the brain are often reflected in the cerebrospinal fluid (CSF), because of the proximity of CSF to the neuronal tissue [11]. The CSF biomarkers Aβ42 and tau can be used to predict future development of AD dementia in patients with MCI [12], [13], [14]. However, to our knowledge there are no fluid biomarkers that can be used to predict the *rate* of the future cognitive decline in persons affected by prodromal AD. Markers associated with increased disease progression rate would be valuable tools both in the clinic and in future therapeutic studies when assessing patients during the pre-dementia stages of AD [11].

In the current study, we characterized the role of CCR2-related chemokines during the pre-dementia stages of AD. Especially we wanted to study whether these chemokines are associated with faster rate of cognitive decline in subjects affected by prodromal AD. Our hypothesis was that concomitant inflammation in the brain might exaggerate AD-associated pathology. The study population consisted of 30 controls and 119 patients with MCI at baseline. All patients were followed over five years and this long clinical follow-up time allowed us to distinguish subjects with prodromal AD from cognitively stable MCI cases, and subjects affected by other forms of dementia. Furthermore, the rate of cognitive decline in individual patients over time could be determined. The levels of the CCR2-ligands CCL2 (MCP-1), CCL11 (eotaxin), CCL13 (MCP-4) and CCL26 (eotaxin-3) were assessed in plasma and CSF obtained at baseline when all individuals were still non-demented.

## Methods

The study population was recruited at the memory disorder clinic, Skåne University Hospital, Malmö, Sweden (demographic data are given in Table 1). At baseline, 119 patients with MCI underwent thorough standard examinations conducted by a trained physician, including neurological, physical and psychiatric examinations. Patients were diagnosed with MCI according to criteria from Peterson et al. [1] including: 1) memory complaint, preferably corroborated by an informant; 2) objective memory impairment adjusted for age and education, as judged by the physician; 3) preservation of general cognitive functioning, as determined by the clinicians judgment based on a structured interview with the patient and a MMSE score greater than or equal to 24; 4) no or minimal impairment of daily life activities; 5) not fulfilling the DSM-IIIR criteria of dementia. Patients with other causes of cognitive impairment, including subdural hematoma, brain tumor, CNS infection, schizophrenia, major depressive episode, and current alcohol abuse were not included. The patients with MCI at baseline were followed clinically until they developed a certain form of dementia or until they had been cognitively stable for >4 years. The patients who during clinical follow-up received a diagnosis of AD had to meet the DSM-IIIR criteria of dementia [15] and the criteria of probable AD defined by NINCDS-ADRDA [16]. After clinical follow-up (median 5 years, range 4–7 years), 52 individuals (44%) remained cognitively stable, 47 patients (40%) developed AD and 20 (17%) developed other forms of dementia.

**Table 1:** Demographic data of included subjects.

Patient characteristics	Controls (n = 30)	Stable MCI (n = 52)	MCI-AD (n = 47)	MCI-other (n = 20)
Age at baseline (years)	72±8	64±9^a^	74±6^b^	72±9^b^
Gender (Male/Female)	13/17	28/24	11/3^b^	13/7
APOE ε4 carrier (%)	23	48	81^a^^, ^ b	30
MMSE at baseline (0–30 p)	29.3±1.0	27.3±1.8^a^	26.7±1.4^a^	27.0±1.6^a^
Annual decrease in MMSE score	−0.01±0.33	−0.21±0.45	3.3±2.5^a^^,^ b	2.6±2.3^a^^,^ b
CSF Tau, pg/ml	283±97	335±203	792±367^a^^,^ b	497±609
CSF P-tau, pg/ml	57±13	63±16	96±29^a^^,^ b	61±31
CSF Aβ42, pg/ml	733±161	555±191^a^	317±98^a^^,^ b	571±179^a^

Data are the mean (± standard deviation) or number (%).Only *P*-values<0.01 are considered significant, because of correction for multiple comparisons (all groups were compared to both controls and stable MCI).

a *P*<0.01 vs Controls.

b *P*<0.01 vs Stable MCI.

Abbreviations: Stable MCI, patients with MCI with stable cognitive functions during a follow-up period of 5.2 years; MCI-AD, patients with MCI who developed Alzheimer's disease during follow-up; MCI-other, patients with MCI who developed other types of dementia during follow-up; CSF, cerebrospinal fluid; *APOE*, apolipoprotein E; MMSE, Mini-Mental State Examination.

The control population consisted of 30 healthy volunteers who underwent lumbar puncture at baseline. Inclusion criteria were the absence of cognitive complaints or symptoms and preservation of general cognitive functioning according to cognitive test (e.g. MMSE, ADAS-cog, clock drawing). Exclusion criteria were active neurological or psychiatric diseases. However, other medical conditions that did not affect cognition were permitted.

At baseline, CSF samples were obtained by lumbar puncture in the L3/L4 or L4/L5 interspace. The samples were then immediately frozen and stored at −80°C pending biochemical analyses. At baseline, non-fasting venous blood for plasma analyses was collected in tubes containing EDTA, which were then centrifuged. Plasma samples were stored in polypropylene tubes at −80°C until biochemical analysis. Both CSF and plasma samples were collected between 9 and 11 am.

### Ethics Statement

All patients gave informed consent to participate in the study. The study was conducted according to the provisions of the Helsinki Declaration and approved by the ethics committee of Lund University, Sweden.

### Assays of CCL2, CCL11, CCL13 and CCL26 chemokines

Levels of chemokines in CSF and plasma were assessed by use of the Human chemokine 9-plex ultra sensitive assay based on the MesoScale technology. The assay is a multiplex type of ELISA technology that relies on electrochemiluminescence. The assays were run according to the manufacturer's protocol (Meso Scale Discovery, USA; http://www.mesoscale.com). Briefly, specific antibodies that coat a working electrode at the bottom of the well capture the molecule of interest. A second antibody labelled with a SULFO-TAG™ binds the molecule of interest. The SULFO-TAG™ emits light upon electrochemical stimulation when a current is applied between the counter electrode and the working electrode, and is registered by a SECTORT™ Imager. Duplicate readings for each standard and sample were averaged and calibration curves were prepared in the supplied assay diluents with a range of 80,000 pg/ml to 0.61 pg/ml, dependent upon the chemokine. Detection limits are defined as 2.5× the standard deviation above the background as calculated by MesoScale Discovery®: CCL2/MCP-1, 3.3 pg/mL; CCL11/Eotaxin, 10 pg/mL; CCL13/MCP-4, 19 pg/mL; CCL26/Eotaxin-3, 14 pg/mL. The intraassay coefficients of variation (CV) ranges were as follows: CCL2: 1.1–6.5%; CCL11: 2.7–3.9%; CCL13: 2.7–5.4%; and CCL26: 1.6–4.9%.

### Statistical analyses

The non-parametric Mann-Whitney *U*-test, with adjustment for multiple comparisons (see Table 1 and Table 2), was used to compare continuous baseline data between the diagnostic groups at follow-up. Pearson's *x* ^2^ test was used for dichotomous variables. The non-parametric Spearman's rank correlation was used for correlation analyses. Multivariate linear regression models (enter) were also used to study the associations between CSF CCL2 and the rate of cognitive decline measured as either annual decrease in MMSE score during follow-up or time until conversion to dementia. The models were adjusted for potentially confounding factors, including age, gender, MMSE total score, education level [participation in post-compulsory school] and *APOE-*ε4 carrier status [carriers of zero or at least one *APOE-*ε4 alleles].The statistical analyses were accomplished with SPSS for Windows, version 18.01.

**Table 2:** The levels of chemokines in cerebrospinal fluid (CSF) and plasma obtained at baseline.

CSF	Controls n = 30	Stable MCI n = 52	MCI-AD n = 47	MCI-other n = 20
CCL2	649±182	676±183	684±194	729±176
CCL11	11±4	12±4	12±5	12±5
CCL13	14±10	12±10	16±13	20±29
CCL26	14±19	33±55	33±48^a^	16±20

Plasma	n = 30	n = 49	n = 42	n = 17
CCL2	149±47	164±44	197±277	172±28
CCL11	411±193	439±193	357±288^b^	451±159
CCL13	271±98	307±108	270±160	339±154
CCL26	41±45	65±156	51±62	30±33

The chemokine levels are given in pg/ml. Only *P*-values<0.01 are considered significant, because of correction for multiple comparisons (all groups were compared to both controls and stable MCI).

a *P*<0.01 vs Controls.

b *P*<0.01 vs Stable MCI.

Abbreviations: Stable MCI, patients with MCI with stable cognitive functions during a follow-up period of 5.2 years; MCI-AD, patients with MCI who developed Alzheimer's disease during follow-up; MCI-other, patients with MCI who developed other types of dementia during follow-up; CSF, cerebrospinal fluid.

## Results

### Subjects and baseline values of chemokine levels

The demographic data and baseline levels of chemokines in CSF and plasma are given in Table 1 and 2. In the control group there were no significant associations between the chemokine levels in CSF or plasma and age, gender, *APOE4* genotype, and baseline MMSE score (data not shown). Only the levels of CCL26 in CSF differed significantly between controls and patients with MCI who subsequently developed AD (p = 0.002) (Table 2).

### Association between CCL2 and cognitive deterioration rate in prodromal AD

**Figure 1:**
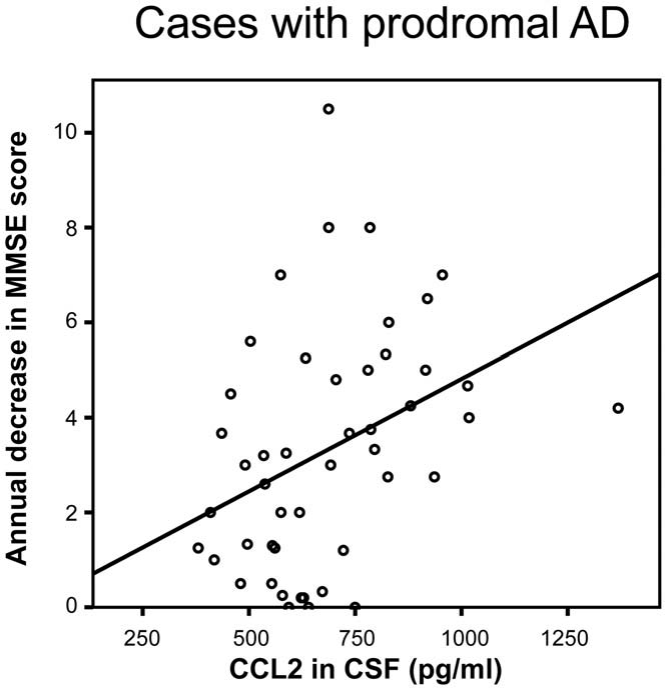
The baseline levels of CCL2 in the cerebrospinal fluid correlated positively with the annual change in MMSE score during clinical follow-up (*r*_s_ = 0.42, p = 0.004).

**Figure 2:**
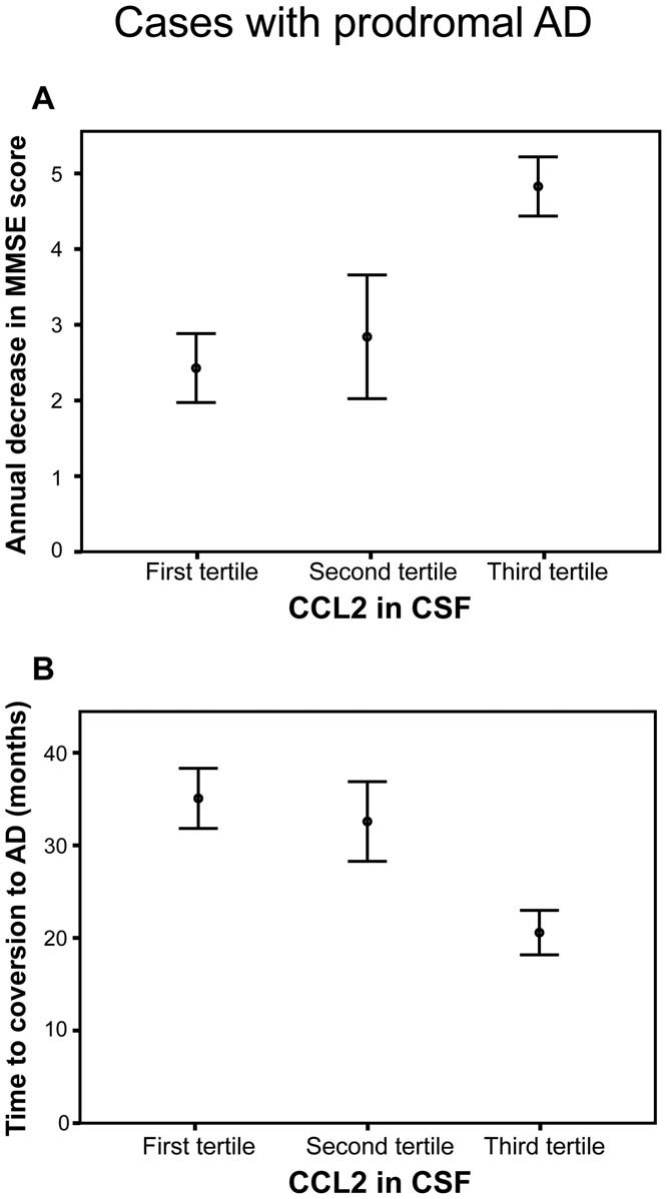
The disease progression rate is increased in cases with prodromal AD who have higher levels of CCL2 in the cerebrospinal fluid at baseline. Panel A shows that prodromal AD cases in the highest tertile of CSF CCL2 at baseline exhibited a significantly higher annual decrease in MMSE score during the follow-up period compared to those in the lowest tertile (p<0.001, Mann Whitney U test). Panel B depicts that prodromal AD cases in the highest tertile of baseline CSF CCL2 exhibited a shorter time period to conversion to dementia compared to those in the lowest tertile (p<0.003, Mann Whitney U test). The CCL2 tertiles were established in the whole MCI cohort (first tertile = 292–584 pg/ml; second terile = 585–756 pg/ml; third tertile = 757–1369 pg/ml). Error bars indicate the standard error of the mean.

When analyzing subjects with MCI who subsequently developed AD (i.e. subjects with prodromal AD) we found that the CCL2 levels in CSF obtained at baseline were significantly associated with a higher annual decrease in MMSE score (*r*_s_ = 0.42, p = 0.004; Figure 1) and with a shorter time to conversion to AD (*r*_s_ = −0.39, p = 0.006). When using multivariate linear regression models and simultaneously including CSF CCL2, age, gender, MMSE total score, education level and *APOE-*ε4 carrier status, we found that only CSF CCL2 was significantly associated with a higher annual decrease in MMSE score during follow-up (p = 0.009) or with a shorter time to conversion to AD (p = 0.016) (Table 3). Thus, higher CSF CCL2 levels are associated with a faster rate of cognitive decline and with a shorter time to conversion to dementia independent of potential confounding factors. Moreover, the annual decrease in MMSE score was higher in cases with prodromal AD who had baseline CSF CCL2 levels in the highest tertile when compared to those in the lowest tertile (Figure 2, p<0.001). Similarly, the time to conversion to dementia was shorter in MCI-AD (i.e. prodromal AD) cases who had CCL2 levels in the highest tertile when compared to those in the lowest tertile (Figure 2, p<0.003). However, there were no correlations between CCL2 levels in *plasma* and annual decrease in MMSE and with time to conversion to AD (p>0.05). CCL11, CCL13 and CCL26 levels in plasma or CSF did not correlate with the measures of disease progression rate (p>0.05).

**Table 3:** Multiple linear regression models for the association between annual change in MMSE score during follow-up and baseline levels of CSF CCL2, including potential confounding factors.

	Model 1	Model 2	Model 3
CCL2 in CSF	0.37 (2.67)*	0.37 (2.64)*	0.39 (2.70)**
Age at baseline		0.02 (0.11)	−0.01 (−0.08)
Gender			0.07 (0.46)
APOE ε4 carrier			0.22 (1.53)
MMSE baseline			0.10 (0.72)
Higher education			−0.12 (−0.76)
R^2^	0.14	0.14	0.20

Values are given as standardized beta coefficients (t-values).

*P<0.05;

**P<0.01.

### Association between CCL2 and cognitive deterioration rate in the whole MCI cohort

**Figure 3:**
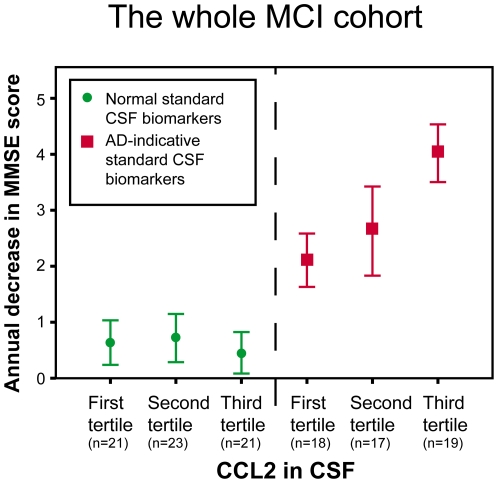
The clinical value of combining baseline levels of CSF CCL2 with standards CSF biomarkers (i.e. Tau, P-tau and Aβ42) in the whole MCI cohort. MCI patients with an AD-indicative CSF biomarker pattern (Tau>350 pg/ml and Aβ42/P-tau ratio <6.5; red squares) declined cognitively during follow-up, because many of these patients developed AD dementia, which was not the case for those with a normal CSF biomarker pattern (green circles). Importantly, the disease progression rate was significantly higher in MCI patients with an AD-indicative CSF biomarker pattern, who also had high CCL2 levels (in the third tertile) when compared to those who exhibited lower levels (in the first tertile) (p<0.05).

The underlying pathology in each individual seeking medical advice due to MCI is not known until extensive clinical follow-up or neuropathology has been performed. However, many studies have shown that CSF Tau, P-tau and Aβ42 can be used to identify those subjects with MCI who have a high risk of developing AD dementia in the future. We therefore investigated whether CCL2 could be used in combination with these standard CSF biomarkers to predict future cognitive decline in the whole MCI cohort. An AD-indicative (pathological) CSF biomarker pattern was defined as Tau>350 pg/ml and Aβ42/P-tau ratio <6.5 as previously described [1]. As shown in Figure 3, patients with MCI who had a normal (i.e. non AD-indicative) CSF biomarker pattern did not decline cognitively over time as a group. In contrast, those with an AD-indicative (pathological) CSF biomarker pattern worsened cognitively over time, because many subjects in this group developed AD dementia during follow-up. Importantly, the subgroup with both an AD-indicative (pathological) CSF biomarker pattern and higher CCL2 levels in CSF at baseline exhibited a significantly faster cognitive decline during follow-up than the other subgroups (Figure 3).

## Discussion

In the present study, we show that higher baseline CCL2 levels in CSF were associated with a faster cognitive decline in patients with MCI who subsequently developed AD. The results indicate that elevated CCL2 levels in the brain might exacerbate the ongoing neurodegeneration in subjects affected by prodromal AD. Interestingly, overexpression of CCL2 levels in the brain of a transgenic animal model of AD expressing mutant APP resulted in a five-fold increase in Aβ deposition [9]. Similarly, in another study CCL2 caused increased microglial accumulation, Aβ oligomerization and cognitive decline in transgenic AD mice [10]. In humans, CCL2 levels are increased in the CSF and brains of demented individuals affected by AD and CCL2 co-localizes with senile plaques [7], [17]. Consequently, CCL2 associated inflammation and microgliosis might have detrimental effects in individuals with altered Aβ metabolism. As a matter of fact, drugs that modify CCL2/CCR2-signaling have already been developed [18] and might be considered for therapeutic trials in prodromal AD.

In the present study, the patients with prodromal AD do not as a group have higher levels of CCL2 when compared to cognitively stable MCI patients or controls. Therefore, CCL2-associated brain inflammation and microgliosis seem to be independent processes, not directly caused by early AD. However, AD-related pathology might anyway be exaggerated when it occurs simultaneously as brain inflammation in the same individual. Recent evidence indicates that many elderly individuals, without dementia or neurological disorders, exhibit increased microglial activation and increased levels of inflammatory markers [19], [20]. Thus, a variable degree of chronic inflammation is often observed in the aged brain. We suggest that the fluctuations in CCL2-levels found amongst the patients with MCI reflect this low-grade immunological activity. It is interesting to note that both epidemiological studies and animal models have demonstrated that inflammatory events (not related to AD), accompanied by elevated chemokine levels, can lead to a significantly accelerated AD progression [21], [22], [23]. It has therefore been suggested that cerebral inflammation, resulting from normal aging or due to inflammatory events, might act in synergy with amyloid beta pathology in cases affected by AD, leading to a steeper cognitive decline [24]. According to the present results, individuals with higher CCL2 levels, reflecting increased brain inflammation, might be more susceptible to AD-pathology than patients with lower levels of inflammatory markers. Furthermore, it is interesting to note in the present study that only CCL2 levels in the CSF, and not in plasma, are associated with an increased progression rate in prodromal AD, indicating that the inflammatory processes important for the disease progression in AD are primarily localized in the brain.

The currently used fluid biomarkers for AD, i.e. CSF Aβ42 and CSF tau, are diagnostic markers that like amyloid PET images change during the presymptomatic stages of the disease. These markers are then quite stable over time [11], and they are not able to predict the future rate of cognitive decline in prodromal AD [12]. Accordingly, CSF Aβ42 and CSF tau should primarily be used as diagnostic markers detecting underlying disease state. Other methods are needed to reflect the stage of the disease and the rate of cognitive decline [11] and the present study suggest that CSF CCL2 is a candidate biomarker for future disease progression rate in prodromal AD.

### Conclusions

Elevated CCL2-signaling in the brain might exacerbate the progression rate of AD-related pathology during pre-dementia stages. Consequently, CCL2-related signaling pathways might be new targets for disease-modifying therapies aiming at slowing down the disease process in patients with prodromal AD. If our results are corroborated by other studies, CSF CCL2 could be used when predicting the future rate of cognitive decline in patients with prodromal AD.
